# Resting-state brain and spinal cord networks in humans are functionally integrated

**DOI:** 10.1371/journal.pbio.3000789

**Published:** 2020-07-02

**Authors:** Shahabeddin Vahdat, Ali Khatibi, Ovidiu Lungu, Jürgen Finsterbusch, Christian Büchel, Julien Cohen-Adad, Veronique Marchand-Pauvert, Julien Doyon

**Affiliations:** 1 Centre de recherche de l’Institut Universitaire de Gériatrie de Montréal, Montréal, Quebec, Canada; 2 Department of Applied Physiology and Kinesiology, University of Florida, Gainesville, Florida, United States of America; 3 McConnell Brain Imaging Center, Montreal Neurological Institute, McGill University, Montreal, Quebec, Canada; 4 Centre of Precision Rehabilitation for Spinal Pain (CPR Spine), University of Birmingham, Birmingham, United Kingdom; 5 Department of Psychiatry, University of Montreal, Montreal, Quebec, Canada; 6 Department of Systems Neuroscience, University Medical Center Hamburg-Eppendorf, Hamburg, Germany; 7 NeuroPoly Lab, Department of Electrical Engineering, Polytechnique Montreal, Montreal, Quebec, Canada; 8 Sorbonne Université, INSERM, CNRS, Laboratoire d’Imagerie Biomédicale, LIB, Paris, France; University of Cambridge, UNITED KINGDOM

## Abstract

In the absence of any task, both the brain and spinal cord exhibit spontaneous intrinsic activity organised in a set of functionally relevant neural networks. However, whether such resting-state networks (RSNs) are interconnected across the brain and spinal cord is unclear. Here, we used a unique scanning protocol to acquire functional images of both brain and cervical spinal cord (CSC) simultaneously and examined their spatiotemporal correspondence in humans. We show that the brain and spinal cord activities are strongly correlated during rest periods, and specific spinal cord regions are functionally linked to consistently reported brain sensorimotor RSNs. The functional organisation of these networks follows well-established anatomical principles, including the contralateral correspondence between the spinal hemicords and brain hemispheres as well as sensory versus motor segregation of neural pathways along the brain–spinal cord axis. Thus, our findings reveal a unified functional organisation of sensorimotor networks in the entire central nervous system (CNS) at rest.

## Abstract 2

This neuroimaging study reveals novel insights into the functional organization of resting-state networks in the brain and spinal cord, such as the contralateral correspondence between the two halves of the brain and spinal cord, and segregation of sensory versus motor neural pathways along this axis.

## Introduction

Spontaneous modulations of the blood-oxygen–level dependent (BOLD) signals from functional magnetic resonance imaging (fMRI) in the absence of any overt task or stimulation have been well characterised in the human brain [1,2]. These slow fluctuations partition the resting brain into temporally synchronised networks of spatially distinct areas, the so-called resting-state networks (RSNs), which mimics the clusters of brain regions that are co-activated during the performance of different sensorimotor and cognitive tasks [2]. Furthermore, several studies have shown functionally specific changes in RSNs following sensory [3] and motor learning [4], hence reflecting the ongoing memory processes related to the acquisition and consolidation of new skills. More recently, RSNs have also been identified within the human spinal cord [5–10]. Specifically, several studies have documented the existence of bilateral as well as unilateral dorsal and ventral networks, which likely represent various sensory and motor processing at the spinal cord level [5–7]. However, it is still unknown whether and how the reported RSNs within each of the brain and spinal cord structures are related to each other (see [11] for a recent description of the RSNs linking the spinal cord and brainstem).

Previous studies have examined functional connectivity between the spinal cord and different brain areas as participants were required to perform various sensory [12] and motor [13] behavioural tasks. For example, our laboratory has reported that the functional interaction between the primary sensorimotor cortex and the anterior cerebellum with the cervical spinal cord (CSC) is dynamically modulated during the early learning stage of a new sequence of finger movements [13]. Likewise, Tinnermann and colleagues have shown that the functional coupling between the prefrontal areas, brainstem, and spinal cord is selectively modulated during nocebo hyperalgesia, whereby pain expectation is increased [12]. Together, these 2 studies provided the first fMRI evidence of a functional correspondence between the brain and spinal cord subregions in the context of a behavioural task. However the intrinsic functional connectivity of the cerebrospinal networks in the absence of any task or external stimulation has never been investigated.

To answer this question, we used a recently developed scanning protocol to acquire functional images of the brain and CSC simultaneously during resting-state periods. The protocol is based on an echo-planar imaging (EPI) sequence, which allows optimisation of the acquisition and shimming parameters for each of the brain and CSC volumes, separately [14,15]. Furthermore, we developed a new processing pipeline for joint analysis of the fMRI signals in these 2 structures using independent component analysis (ICA), as well as the region of interest (ROI)–based functional connectivity method. We hypothesised a stronger resting functional connectivity between each hemispinal cord and contralateral brain areas compared to ipsilateral areas, consistent with the known decussation of major efferent and afferent pathways between these structures [16]. In addition, based on the organisation of spinal cord anatomical connections, both intrinsic and with the supraspinal structures [17], we predicted that dorsal and ventral regions of the spinal cord would show distinct patterns of resting-state connectivity with the sensory and motor brain areas, respectively. Finally, we investigated the interrelationships between spinal cord and the consistently reported brain RSNs in humans [18].

## Results

**Fig 1:**
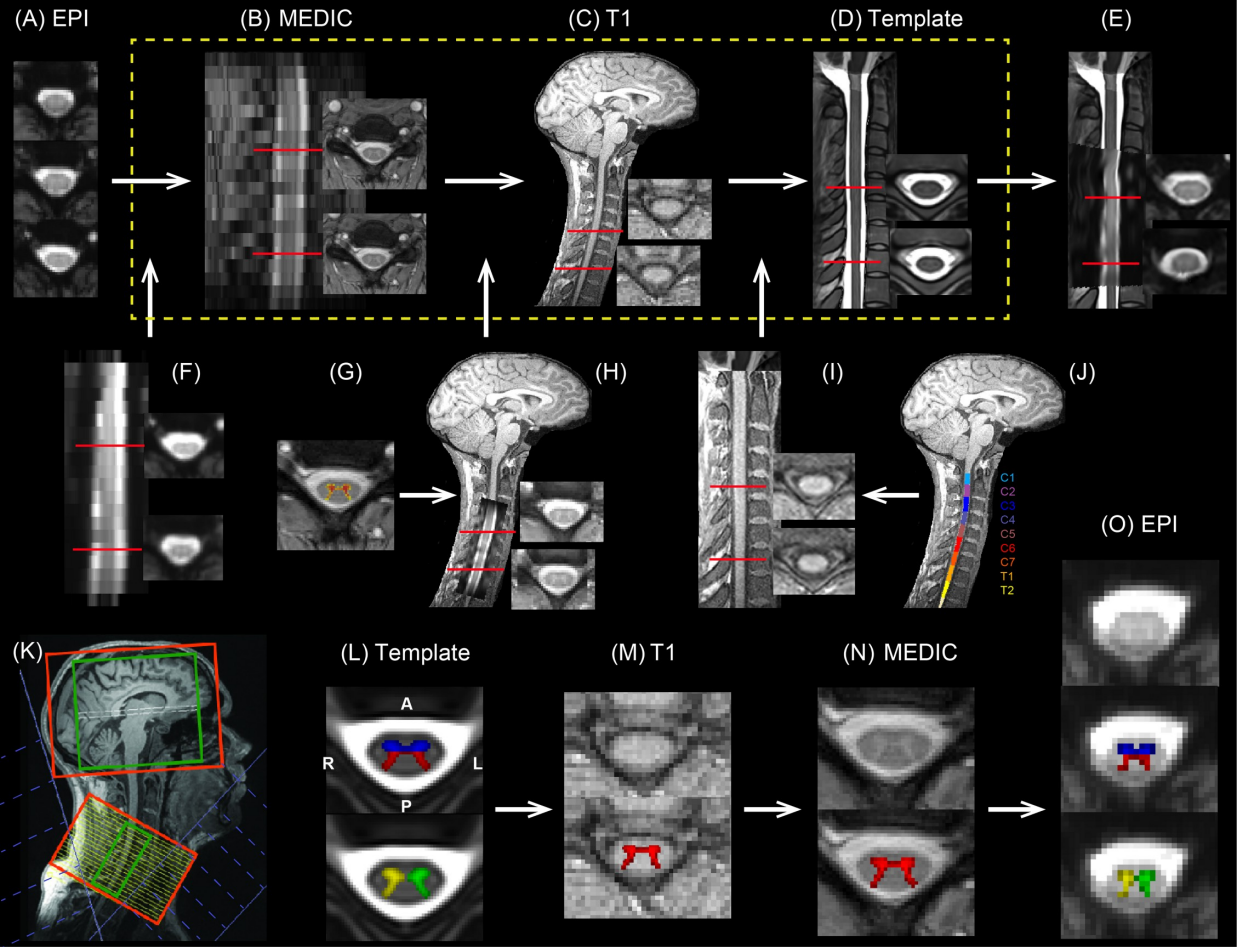
The processing pipeline for the spinal cord ROI analysis. (A–D) The functional EPI images (A) were sequentially registered to the T2*-weighted MEDIC image (B), then to the T1-weighted image (C), and finally to the MNI-Poly-AMU template (D). Panels show representative horizontal and sagittal slices for a sample participant. (E) Shows the result of combined transformations, i.e., the EPI spinal cord data registered to and overlaid on the MNI-Poly-AMU template. (F) Shows the first transformation, i.e., the EPI spinal cord data registered to the MEDIC image space. (G) Shows the grey matter segmentation of the MEDIC spinal cord image used to improve local registration based on the dictionary approach. (H) Shows the second transformation, i.e., the MEDIC image registered to the participant T1-weighted space. (I) Shows the third transformation, i.e., the T1-weighted image registered to the MNI-Poly-AMU template. (J) Shows the segmentation of T1-weighted image to different vertebral levels (C1 to T2), used in the registration to the template. (K) Outline of the brain (upper) and the spinal cord (lower) FOVs for EPI scans (red boxes), and the shimming volumes (green boxes) overlaid on the T1-weighted image. The lower FOV covers the spinal cord between C4 and T1 vertebral levels. Blue boxes show the two saturation pulses in a V-shaped configuration. (L) Blue, red, yellow, and green masks, respectively, show the segmentation of ventral, dorsal, right, and left spinal cord ROIs defined in the MNI-Poly-AMU template. (M–O) The spinal cord ROIs were sequentially registered to the T1-weighted image (M), and then to the T2*-weighted MEDIC image (N), and finally to the EPI image space (O), based on the inverse transformations found in panels A–I. The red areas in (M) and (N) show the registered grey matter segmentation from the template to each image space. C1, first cervical; EPI, echo-planar imaging; FOV, field of view; MEDIC, multi-echo data image combination; ROI, region of interest; T2, second thoracic.

We first investigated the laterality in functional connectivity between the brain hemispheres and cervical spinal hemicords (halves). For this analysis, we calculated the average signal in the left and right sides of the spinal grey matter (see Fig 1 for definition of ROIs and registration to the spinal cord template) and identified brain areas that are correlated with each spinal hemicord during resting-state periods.

**Fig 2:**
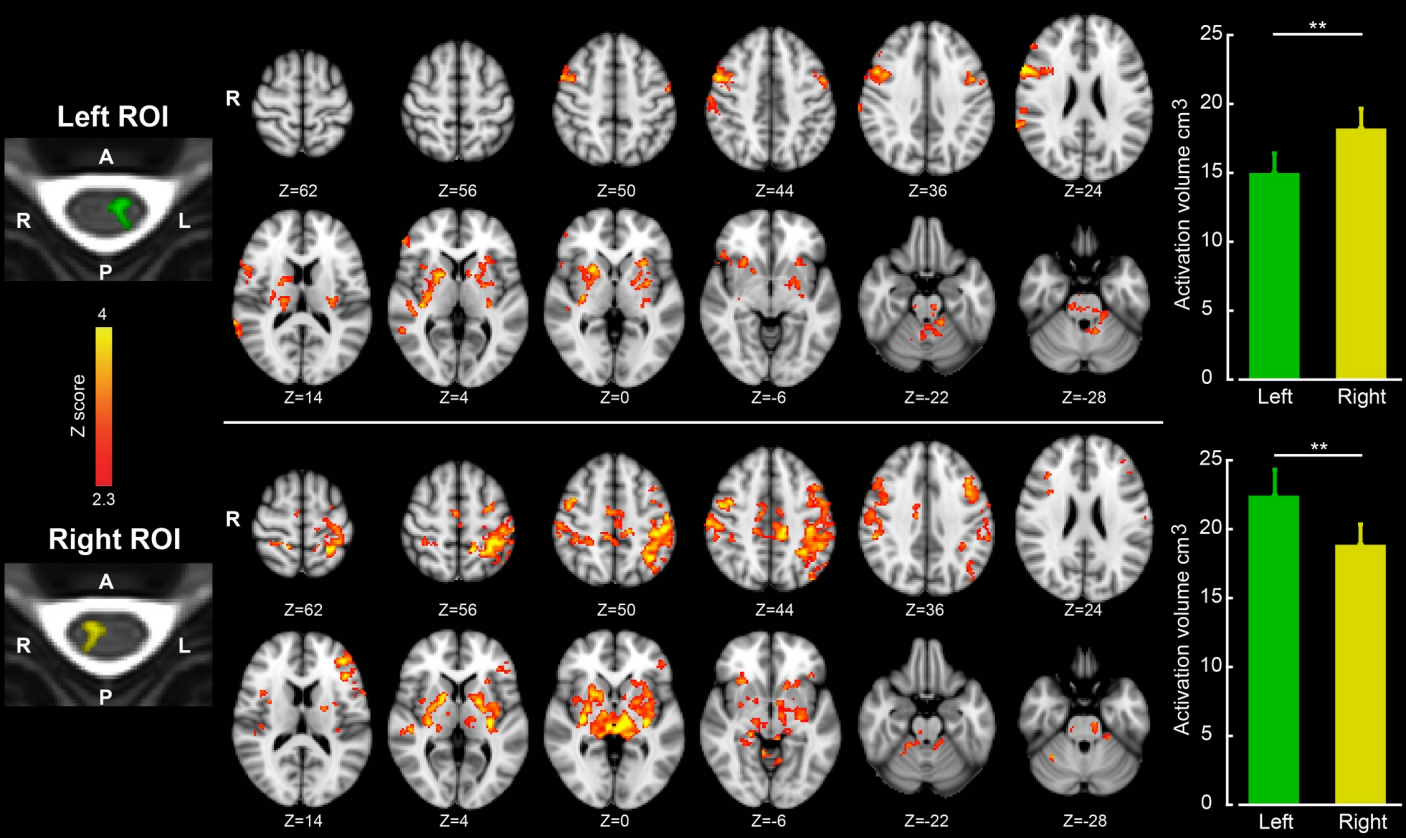
The spinal hemicords are preferentially connected to the contralateral cerebral cortex at rest. The left column shows the location of spinal ROIs, and on the right their associated brain functional connectivity maps are presented. As shown, the left (top row) and right (bottom row) spinal ROIs are mostly correlated with the right and left cerebral cortex, respectively. Color-coded activation maps indicate z-score values and are corrected for multiple comparisons using GRF, *p* < 0.05; Z-coordinates report MNI space. Bar plots show the average activation volume in the left (green) and right (yellow) brain hemispheres correlated to the left spinal ROI (top panel) and right spinal ROI (bottom panel). As shown, the left spinal ROI is correlated with a larger volume in the right brain hemisphere (**p < 0.01), while the right spinal ROI is correlated with a larger volume in the left brain hemisphere (**p < 0.01). Error bars represent SEM. The numerical data used in this figure are included in S1 Data. GRF, Gaussian random field; MNI, Montreal Neurological Institute; ROI, region of interest.

Fig 2 shows the brain regions with significant functional connectivity to the left and right CSC (cluster-level *p* < 0.05, corrected for multiple comparisons using Gaussian random field [GRF] theory). As shown, the BOLD signal within the left and right spinal ROIs are correlated with several brain areas, including primary somatosensory and motor cortices, supplementary motor area, premotor cortex, posterior partial cortex, Broca’s area, insula, putamen, thalamus, and cerebellum. Notably, it appears that the right CSC is more correlated with cortical areas in the left hemisphere, and vice versa. To specifically test this hypothesis, we examined the brain activation volume (brain voxels with significant correlation with spinal time series; *p* < 0.01) functionally connected to each spinal cord ROI in each participant. We then measured the activation volume in each brain hemisphere separately, based on which a laterality index was calculated for each spinal hemicord (see Methods). This analysis revealed a significant interaction in functional connectivity between the sides of the spinal cord and those of the cerebral cortex (*p* < 0.001). Specifically, with respect to the left spinal cord, the right cerebral hemisphere revealed significantly larger volume of activation than the left hemisphere (Fig 2 top bar plot; paired-sample *t* test, left ROI: *t*_23_ = 3.44, *p* = 0.002). The laterality index was significantly less than zero (mean value: −10.97; *p* < 0.001), hence indicating a larger activation volume in the right cerebral hemisphere. Inversely, the volume of activation in the left cerebral hemisphere was significantly larger than that in the right hemisphere, when the right spinal cord was used as predictor (Fig 2 bottom bar plot; paired-sample *t* test, right ROI: *t*_23_ = 2.87, *p* = 0.009). This time, however, the laterality index was significantly greater than zero (mean value: 8.1; *p* = 0.006), indicating a preference in laterality toward the left cerebral hemisphere activation. Hence, this analysis confirmed stronger functional connectivity between the contralateral brain and spinal cord sides on a per-individual basis.

In a follow-up analysis, we repeated the analysis reported in Fig 2 but this time included both the segmented left and right sides of the spinal grey matter time series in the same regression model so that only part of the variance attributed exclusively to each side was used to predict brain connectivity (see Methods for more details, [4]). In other words, the shared variance between the two sides of the CSC was not used to estimate the connectivity of each hemicord with the brain areas. The results of this analysis reported in S1 Fig revealed that BOLD signal within the left spinal cord is exclusively correlated with that of brain areas in the right hemisphere. In contrast, activity within the right spinal cord correlated significantly with brain activities in the left hemisphere. The observed left-right correspondence in functional connectivity of the spinal cord and brain is consistent with the anatomical connectivity of the main efferent and afferent pathways, both decussating at the brainstem level [17].

**Fig 3:**
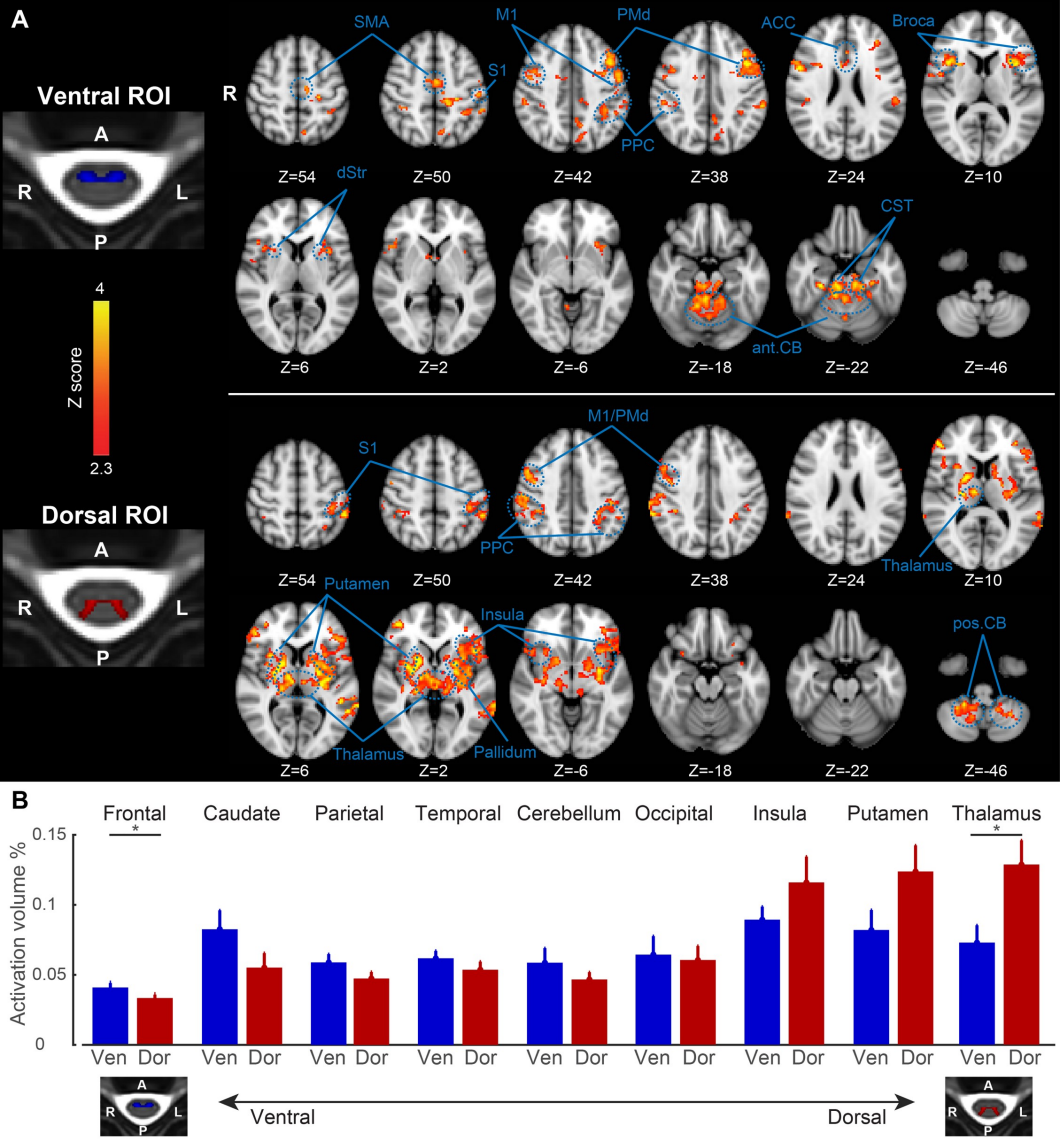
Distinct resting-state brain connectivity associated with the ventral and dorsal horns of the spinal cord. (A) The ventral horn (top row) is significantly correlated with bilateral M1, dorsal premotor cortex, supplementary motor area, anterior cingulate cortex, Broca’s area, S1, posterior parietal cortex, anterior dorsal striatum, anterior cerebellar cortex, and corticospinal tract. On the other hand, the dorsal horn (bottom row) is significantly correlated with bilateral S1, posterior parietal cortex, insula, thalamus, putamen, pallidum, M1, dorsal premotor cortex, and the posterior cerebellum. Color-coded activation maps indicate z-score values, corrected *p* < 0.05. (B) Key brain areas sorted based on their strength of functional connectivity to the ventral and dorsal horns of the spinal cord. Bar plots show the percent volume of activation related to the ventral (blue bars) and dorsal (red bars) spinal horn in different brain areas. From left to the right, the brain areas are sorted based on their functional connectivity preference to the ventral horn (the most left) or to the dorsal horn (the most right). The percent volume for each brain area is calculated by dividing the active volume (*p* < 0.01) to the total volume of that area. As shown, the frontal lobe, caudate, and parietal lobe tend to be more synchronised with the ventral horn activation, while the thalamus, putamen, and insula tend to be more synchronised with the dorsal horn activation at rest. The 9 brain areas are defined from the parcellation of brain in the MNI structural atlas. *p < 0.05. The numerical data used in this figure are included in S1 Data. ACC, anterior cingulate cortex; ant.CB, anterior cerebellum; CST, corticospinal tract; Dor, dorsal; dStr, dorsal striatum; MNI, Montreal Neurological Institute; PMd, dorsal premotor cortex; pos.CB, posterior cerebellum; PPC, posterior parietal cortex; SMA, supplementary motor area; Ven, ventral.

Next, we examined the brain functional connectivity associated with the ventral and dorsal horns of the spinal cord (see Fig 1L–1O for definition of ROIs). Again as in Fig 2, to capture all brain areas correlated with the dorsal and ventral horns, in this analysis we employed 2 separate regression models to estimate the whole brain functional connectivity maps associated with each ROI. Fig 3A shows, at the group level, the brain areas that are significantly correlated with the ventral horn of the CSC at resting-state condition, including bilateral frontal motor areas (M1, dorsal premotor cortex, supplementary motor area, and anterior cingulate cortex), Broca’s area (Brodmann area 44), somatosensory areas (S1, and posterior parietal cortex), anterior dorsal striatum, anterior cerebellar cortex (lobules I–IV), and corticospinal tract. On the other hand, resting-state activity in the dorsal horn was significantly correlated with that of bilateral parietal areas (S1, and posterior parietal cortex), insula, thalamus, putamen, pallidum, M1, dorsal premotor cortex, and the posterior cerebellum (lobule VIII). These findings suggest that the CSC shows functionally specific resting-state connectivity with different parts of the cerebellum and basal ganglia according to anteroposterior segregation, where anterior and posterior regions in these structures are more correlated to the ventral and dorsal horns, respectively. Additionally, our results demonstrate that resting-state activity in some frontal motor areas such as supplementary motor area and anterior cingulate cortex mainly correlated to that of the ventral horn, while thalamus and insula are mainly correlated to the dorsal horn. Finally, resting-state BOLD signal in some areas such as somatosensory partial areas seems to be correlated to that of both ventral and dorsal horns.

In a follow-up analysis, we segmented each individual’s brain image based on the Montreal Neurological Institute (MNI) structural atlas into 9 key areas [19,20], including frontal, parietal, temporal, occipital, insula, putamen, caudate, thalamus, and the cerebellum. This anatomical parcellation allowed us to compare the preference in functional connectivity of main brain areas with respect to the ventral and dorsal horns of the spinal cord. Hence, we calculated the percent volume of activation (significantly correlated with the dorsal and ventral horns, *p* < 0.01) in each brain parcellation on a per-individual basis. Fig 3B reports the results of this analysis, where different brain areas are sorted based on their functional connectivity preference to the ventral and dorsal horns, from left to right. On one end, the frontal lobe showed greater correlation with the ventral horn compared to the dorsal horn (*t*_23_ = 1.99, *p* < 0.05). On the other end, however, thalamus showed greater functional connectivity with the dorsal horn compared to the ventral horn (*t*_23_ = 2.66, *p* < 0.05). All the other brain areas showed a partial (nonsignificant) preference to each spinal horn. Note that this analysis gives an overall volume measure across the entire brain region, so it is insensitive to differences in the location of activation within a region (e.g., in the cerebellum or basal ganglia).

Next, in order to obtain a more detailed representation of the CSC–brain functional connectivity, we examined the brain connectivity associated with each CSC quadrant, i.e., the left and right dorsal, as well as the left and right ventral horns. S2 Fig shows the brain areas with significant BOLD signal correlation with the left and right ventral horn (cluster-level *p* < 0.05, corrected). Consistent with the results reported in Fig 3A, the ventral quadrants are significantly correlated with frontal motor areas, including supplementary motor area, M1, premotor cortex, Broca’s area, S1, posterior parietal cortex, and anterior cerebellum (lobule I–IV). As demonstrated in the figure, there is a tendency for the correlation of ventral horn quadrants with the contralateral side of the brain (except for the cerebellum). On the other hand, S3 Fig shows the brain areas that are significantly correlated with the left and right dorsal horn (cluster-level *p* < 0.05, corrected). As shown, the dorsal quadrants are significantly correlated with S1, posterior parietal cortex, ventral premotor cortex, insula, thalamus, ventral posterior putamen, pallidum, and posterior cerebellum (lobule VIII). Again, there is a tendency for the correlation of dorsal horn quadrants with the contralateral side of the brain (except for the cerebellum).

In order to further examine and visualise the contralateral relationship between the sides of the spinal cord quadrants and the brain hemispheres, in a follow-up analysis the left and right counterparts in S2 and S3 Figs were entered into a single regression model to account for the shared variance between the left and right sides. Here again, we found that the left and right quadrants were exclusively correlated to the right and left cortical areas, respectively (S4 Fig).

Furthermore, we investigated brain functional connectivity associated with the grey commissure in the cervical cord. The grey commissure is a transverse band of grey matter surrounding the spinal central canal that, together with the anterior white commissure, connects the 2 spinal hemicords (green ROI in S5 Fig). This analysis revealed a significant functional connectivity between the grey commissure and several bilateral brain areas, including basal ganglia (putamen, pallidum, and caudate), thalamus, insular cortex, and secondary somatosensory cortex (S5 Fig).

**Fig 4:**
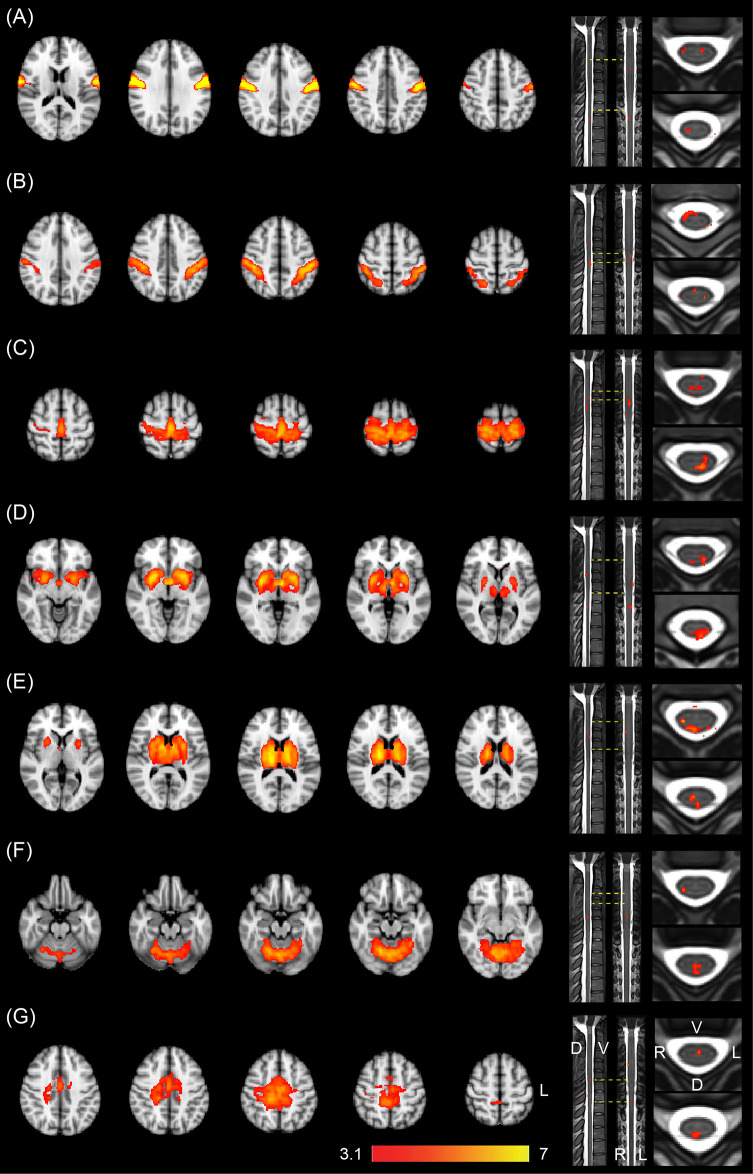
Joint ICA of brain and spinal cord RSNs. ICA revealed 7 distributed networks that include both brain and spinal cord clusters. At the brain level, these networks include bilateral primary motor cortex (A), somatosensory cortex (B), dorsal sensorimotor (C), basal ganglia (D), thalamus (E), cerebellum (F), and the supplementary motor area (G). At the spinal cord level, they mainly cover the ventral or medial regions (A, B), the dorsal regions (D, E, F), or both dorsal and ventral regions of the cervical cord (C, G). Each spinal cord component is presented, from left to right, in sagittal, coronal, and axial planes. Color-coded activation maps indicate z-score values, corrected *p* < 0.05. The numerical data used in this figure are included in S1 Data. D, dorsal; ICA, independent component analysis; L, left; R, right; RSN, resting-state network; V, ventral.

Finally, in order to examine the relationship between brain and spinal cord RSNs, we performed a joint ICA by combining the simultaneous brain and spinal cord resting-state fMRI data together (see Methods for details). This analysis resulted in 15 significant group-level components (corrected, *p* < 0.05), in which their associated brain maps were similar to or part of the consistently reported brain RSNs in the literature [18,21], and their corresponding time series fluctuated mostly in the neural activity-related frequency band of the resting-state BOLD signal (0.01 to 0.1 Hz [2]; see Methods for details). Seven of the 15 components revealed the existence of distributed networks that contained both brain and spinal cord clusters (Fig 4). Their associated brain maps corresponded generally to components of the motor, somatosensory, and subcortical sensorimotor RSNs. They covered 4 cortical brain regions, including the primary motor (along the central sulcus; Fig 4A), somatosensory (along the postcentral sulcus; Fig 4B), the dorsal sensorimotor (Fig 4C), and the supplementary motor area (Fig 4G), as well as 3 subcortical regions, including the basal ganglia (Fig 4D), the thalamus (Fig 4E), and the cerebellum (Fig 4F).

Each of these sensorimotor components showed co-activation with several clusters at different levels of the CSC. As shown in Fig 4, the primary motor and somatosensory components mainly showed co-activation with the ventral and medial parts of the spinal cord, while the subcortical components (basal ganglia, thalamus, and cerebellum) were mainly co-activated with the dorsal regions of the spinal cord. Furthermore, the dorsal sensorimotor and supplementary motor area components were associated with activity in both dorsal and ventral parts of the spinal cord.

The remaining 8 networks resembled other widely reported brain RSNs but contained no or only very small spinal cord co-activation (S6 Fig). These networks included the right and left executive control (S6A and S6B Fig), medial and lateral visual (S6F and S6H Fig), auditory (S6E Fig), and the anterior and posterior parts of the default mode network (S6C, S6D and S6G Fig). Among these, the left and right executive control as well as the anterior and posterior parts of the default mode network were co-activated with a small cluster in the spinal cord (spinal cord activation volume < 0.15 cm^3^), while the other networks did not include any significant cluster in the spinal cord. Comparing Fig 4 and S6 Fig clearly indicates that the spinal cord subregions were mostly co-activated with brain networks that are commonly associated with motor and somatosensory functions during resting-state periods.

## Discussion

In this study, we used a unique scanning protocol to acquire functional images of the brain and CSC simultaneously during resting-state periods and developed a new processing pipeline for joint analysis of the fMRI signals in these two structures using ICA and ROI-based functional connectivity approaches. Using these techniques, we provide the first neuroimaging evidence for the existence of resting-state functional networks spanning both the brain and spinal cord structures. Importantly, our results provide functional evidence for dominance of contralateral projections, as well as dorsoventral segregation of spinal cord connectivity with the somatosensory and motor brain areas. In summary, our findings reveal a close relationship between spontaneous activities of different brain areas with musculoskeletal afferent and efferent intrinsic activities, which may reflect a functional property of the entire central nervous system (CNS) at rest.

Specifically, the functional connectivity analyses using the left/right hemicords and the spinal quadrants as ROIs showed that the resting-state BOLD signal in the left spinal cord is primarily correlated to brain areas in the right hemisphere, and vice versa. This shows a strong influence of contralateral projections between the spinal cord and cerebral hemispheres in their spontaneous activity and is consistent with the decussation of major afferent and efferent pathways [16]. A recent study investigating the CSC-brainstem RSNs did not observe such a contralateral organisation of the functional connectivity [11]. This discrepancy may be related to the fact that, for some sensory pathways such as the dorsal column-medial lemniscus pathway, the synaptic relay at the brainstem level is located on the same side of the first sensory neurons. Also for the motor pathway, most of the corticospinal neurons decussate in the pyramids of the lower medulla. Hence, in contrast to the cerebral hemisphere, a strong ipsilateral connectivity between the brainstem and spinal hemicords is expected. Moreover, examination of the overall bilateral cerebral activity revealed that a larger volume of cerebral cortex was correlated with the right than with the left CSC (40.88 cm^3^ and 33.21 cm^3^, respectively), which might be related to the fact that all participants in our study were right-handed.

Furthermore, the dorsal and ventral horns of the spinal cord were functionally connected to distinct areas of the brain during resting-state periods. Interestingly, the ventral and dorsal horns were preferentially correlated with different subregions within the cerebellum and striatum; the ventral horn was functionally linked with the anterior cerebellar cortex (lobules I–IV) and the rostrodorsal putamen, while the dorsal horn was linked with the intermediate region of the posterior cerebellum (lobule VIII), as well as the caudodorsal and ventral putamen and pallidum. Previous human neuroimaging studies have demonstrated a dorsal/ventral distinction in the striatum, where the dorsal putamen and caudate were mainly co-activated with the anterior cingulate cortex, insula, primary motor cortex, and premotor areas, while the ventral striatum was co-activated with bilateral medial temporal lobe, amygdala, and hippocampus [22]. In another study, rostrodorsal putamen activation was more correlated with middle and superior frontal gyri as well as anterior cingulate cortex, while the caudal parts of the putamen were more correlated with sensorimotor cortical areas [23]. Consistent with these findings, we found that the rostrodorsal part of putamen was mainly correlated with the ventral horn, while the other parts of putamen (including the ventral and caudal subregions) were largely correlated with the dorsal horn. These observations are generally in line with the model of the tripartite division of the striatum based on its cortical inputs, in which the motor and associative cortical areas project to the dorsal striatum, whereas the limbic areas project to the ventral striatum [22,24,25].

The presence of sensorimotor homunculi in the anterior lobe (lobules I–VI) and lobule VIII of the cerebellar cortex has also been documented [26,27]. Meta-analysis of fMRI data shows that both the anterior lobe and lobule VIII are activated during various sensorimotor tasks, while working memory paradigms activate lobules VI, Crus I, and VIIIA [28,29]. Moreover, the dorsal and medial olivary nuclei, as well as the dorsal and ventral spinocerebellar tracts, project to the anterior lobe and lobule VIII in cat (see [30], for a review). Consistent with these findings, our results show a significant functional link between the spontaneous activities of the anterior lobe (lobules I–IV) and lobule VIII with the spinal cord. In addition, they suggest a functional dissociation of anterior lobe and lobule VIII of the cerebellum with respect to the ventral and dorsal horns of the CSC, respectively. Interestingly, thalamic activity was mainly correlated with that of the dorsal horn, consistent with a role of the thalamus in early sensory processing as well as with the thalamus being the main target of somatosensory afferent pathways from the spinal cord [31]. Finally, as predicted by the organisation of afferent and efferent projections, frontal cortical areas showed greater functional connectivity to the ventral horn, while insular cortex was mainly correlated with the dorsal horn (Fig 3). Parietal cortex (S1 and posterior parietal lobule), however, showed significant functional connectivity to both ventral and dorsal horns of the spinal cord during resting-state periods. This finding is consistent with significant descending projections to the ventral horn of the spinal cord (mainly to spinal premotor interneurons) from the parietal cortex [32,33].

Also, our results showed that the grey commissure or the central grey of the spinal cord (corresponding to the Rexed lamina X) is significantly correlated with bilateral basal ganglia, thalamus, and insular cortex (S5 Fig). The central grey receives somatic and visceral pain afferents from both C and Aδ fibres, contains decussating axons to other hemicord, and is involved in nociception, visceral pain, mechanoreception, and modulation of the motor output [34–36]. These proposed functions are consistent with the observed pattern of functional connectivity to central regions of the brain as stated earlier.

Notice that some brain areas, such as the cerebellum, only appeared in the left or right ventral or dorsal quadrants (S2 and S3 Figs). This pattern of results may be related to the low signal-to-noise ratio (SNR) and high variability in the spinal cord resting-state data, as described subsequently. Hence, averaging the spinal cord time series in a smaller ROI related to each quadrant may have resulted in a reduced detection power as compared with averaging the signal in the entire ventral or dorsal horn. Indeed, when we lowered the significance threshold for the statistical tests, we were able to detect a more widespread brain network, which resembled similar contralateral areas for the left and right spinal quadrants.

In addition, to extract interconnected brain and spinal cord networks during the resting-state periods, we developed a joint ICA approach. This analysis identified 15 CNS networks, the brain components of which closely resembled the consistently reported resting-state brain RSNs [18]. Seven of these networks encompassed significant activation clusters in the spinal cord. Consistent with the results of seed-based analysis, the CSC networks covering mostly the dorsal and ventral horns were synchronised with the brain networks commonly associated with the somatosensory and motor functions, respectively. Although still speculative, this suggests that the spontaneous activities of the afferent and efferent signals during resting-state periods might keep the brain and spinal cord connections functionally active and follow the same organisation as that in the active task performance. Consistent with this view, several studies have reported disrupted connectivity within and between brain RSNs following spinal cord injury [37,38], suggesting that specific nodes within the spinal cord may play an important role in the cerebral internetwork connectivity.

fMRI of the human spinal cord is very challenging because of a multitude of factors, including the small diameter of the spinal cord, susceptibility artifacts due to local magnetic field inhomogeneities, pulsatile cerebrospinal fluid (CSF) surrounding the spinal cord, and motion artifacts due to the proximity to thorax, lungs, and neck muscles. The specific gradient echo EPI sequence developed previously [14] and employed here allowed simultaneous acquisition of the brain and spinal cord volumes using different parameters optimised for each structure. The combined acquisition sequence and the advanced shim procedures (including dynamic shim update, and z-shimming) [14,15] enabled us to acquire spinal cord images with low distortion due to magnetic susceptibility artifacts and with high SNR while keeping the spatial resolution at a reasonable range (1.2 mm in-plane) and covering the whole brain within a reasonable acquisition time (total repetition time [TR] = 3,050 ms). Also, the use of saturation pulses around the spinal field of view (FOV), as well as flow rephasing gradient pulses, allowed us to minimise ghosting and signal variations related to pulsatile CSF. Furthermore, in our analysis we carefully modelled and removed the effects of physiological noise in the BOLD signal by including slice-wise physiological noise modelling (PNM) regressors extracted from the recorded cardiac and respiratory signals [39], motion correction parameters, and the average white-matter and CSF signals as confound in the general linear model (GLM). This combination of the image acquisition and analysis considerations allowed us to overcome the previously mentioned technical challenges in examining the resting-state brain and spinal cord networks through a simultaneous recording approach.

Several studied have examined the pattern of functional connectivity within the human spinal cord using fMRI [5,7,40]. Some of these studies have reported between-segmental functional connectivity across the ventral and dorsal horns [40], while others have reported a restricted spatial extent rostro-caudally [5,10]. With respect to the within-segmental pattern of connectivity, the spinal networks are usually divided into ventral and dorsal components [6], with greater bilateral connectivity within the ventral than dorsal component [5]. Moreover, studies employing ultra-high field strengths (7T MRI) have demonstrated high reproducibility and robustness of the spinal cord RSNs within individuals [6,41]. Our results using the joint ICA of the brain and spinal cord signals also confirm these previous findings by demonstrating within- and between-segmental connectivity patterns in several networks. Particularly, the identified spinal cord networks can roughly be separated into bilateral ventral [Fig 4A and 4B], bilateral dorsal [Fig 4D, 4E and 4F], unilateral within-segmental [Fig 4C], and medial between-segmental components [Fig 4G]. Furthermore, the striatal, cerebellar, and thalamic networks were mostly synchronised with the dorsal spinal networks, while the sensorimotor cortical areas were functionally connected to the ventral spinal networks, and supplementary motor area was associated with a medial spinal cord component. These results demonstrate a close relationship between the brain and spinal cord RSNs, which together form a multilevel representation of the CNS RSNs. It is worth noting, however, that several factors—including differences in the imaging acquisition parameters, SNR, and sensitivity to common confounds between the brain and spinal cord—might have impacted the results of our joint ICA analysis, leading to disproportionate influence of one of these structures on the extracted joint components (probably toward more pronounced brain components due to higher SNR in the brain). Future studies are thus required to investigate the impact of these parameters on the joint ICA results.

Several research groups have investigated changes in functional connectivity of the spinal cord networks following or during different sensory stimulations [12,13,40,42]. One study has shown that interindividual differences during noxious heat stimuli can be explained by connectivity strengths in a network of brainstem and spinal cord regions [42]. In another study, thermal stimulation modulated spinal cord connectivity in a bilateral dorsal spinal network [40]. Interestingly, Tinnermann and colleagues [12] have recently shown that high-level cognitive information such as medication monetary value can modulate the functional connectivity between the spinal cord and brainstem during nocebo hyperalgesia. In another study, we have recently shown that the connectivity of the brain and spinal cord regions during a motor sequence learning task is dynamically modulated by the amount of learning [13]. These studies, as a whole, examined the brain and spinal cord connectivity during a functional task, or functional connectivity within the spinal cord during resting-state conditions. However, no study so far has investigated the pattern of connectivity between the brain and spinal cord during resting-state periods. Thus, the present study provides not only evidence for the functional connectivity at rest, which spans the lower and upper levels of the CNS, but also a methodological prescription of how the RSNs between these structures can be examined.

Assessing the functional association of the brain and spinal cord simultaneously is extremely challenging in humans, and, beside a few studies mentioned earlier, it is usually investigated using electrophysiological approaches that target specific sensory inputs using peripheral nerve stimulation, or using transcranial magnetic stimulation to target outputs of the brain [43]. However, it is not possible, using these techniques, to investigate the basic activity of the CNS without conditioning stimuli that perturb the CNS activity per se, and besides, identifying the precise source of supraspinal influence on spinal circuits is very difficult. The resting-state functional connectivity method presented here, on the other hand, can provide a new complementary approach to study the modulation of different cortical areas on the spinal cord circuits in vivo.

Our findings are likely to have direct applications in identifying changes in functional organisation of neural circuits in various movement disorders that impact both the brain and spinal cord structures. In these cases, assessing brain–spinal cord connectivity can provide invaluable information regarding the disease diagnosis and progression, as well as the effectiveness of various clinical interventions. In sum, our findings reflect a functionally relevant organisation of afferent and efferent signals across the CNS at rest.

## Methods

### Participants

The ethics committee at the Centre de Recherche de l'Institut Universitaire de Gériatrie de Montréal reviewed and approved the study (protocol number: CMER-RNQ 15-16-06), which adhered to the Declaration of Helsinki. All participants signed an informed consent prior to participating in the study and were debriefed and compensated at the end of the experiment. Twenty-eight young, right-handed, healthy adults were selected to take part in this study based on the following exclusion criteria: a history of neurological and psychiatric diseases, any motor-system complication, use of medication other than contraceptives, and presence of any MRI-incompatible object in the body. The data from one participant were excluded from the analysis due to excessive head/neck movement in the scanner (more than 0.2 mm and 0.005 rad of mean translation and mean rotation, respectively, compared to all other participants, who had mean translation and mean rotation below 0.05 mm and 0.001 rad, respectively). Additionally, the data from 3 other participants were excluded since we did not cover their whole brain in the functional scan (for these 3 participants, the most dorsal fully covered axial slice was at Z = 54 in the MNI space, while in the rest of the participants, the brain was fully covered up to Z = 66 in the MNI space). As such, the final sample considered for analysis consisted of 24 participants (13 females, mean age = 25.1 years).

### MRI data acquisition

A 3T TIM Trio Siemens scanner (Siemens Medical Solutions, Erlangen) equipped with a 12-channel head coil paired with a 4-channel neck coil was used for the imaging. To investigate the functional connectivity between the brain and the spinal cord, a specific EPI sequence was used to enable simultaneous acquisition of fMRI data (BOLD contrast) from the brain and spinal cord [12]. Details of the MRI pulse sequence can be found elsewhere [14,15].

Participants laid on the scanner table in a supine position with their head and neck fully supported using foam pads to minimise their motion. To avoid excessive movements of the body, participants’ shoulders were strapped to the table using Velcro bands. Participants were placed in the scanner such as that the mid-chin level was located in the scanner’s isocentre, corresponding to the vertebral level C2-C3 (second and third cervical) on the axial plane.

For the EPI measurements, 43 slices were acquired in ascending order divided into two subvolumes (FOVs) (Fig 1K). The upper FOV included 33 axial slices oriented along the anterior-commissure–posterior-commissure axis to cover the whole brain (for some participants the very top of the brain [MNI coordinate: Z > 68] could not be covered). The lower FOV included 10 slices oriented approximately perpendicular to the spinal cord at the C6 level, covering the spinal cord between C4 and T1 (first thoracic) vertebral levels (C4, C5, C6, C7, T1; see Fig 1K).

This specific gradient echo EPI sequence allowed us to adjust the MRI acquisition parameters separately for each FOV based on the resolution/coverage needs for each of the brain and spinal cord. For the brain, subvolume imaging parameters were as follows: FOV = 220 × 220 mm^2^; in-plane resolution = 2 × 2 mm^2^; slice thickness = 3.5 mm with no gap; echo time (TE) = 30 ms; bandwidth = 1,514 Hz/Px; echo spacing = 0.74 ms; and flip angle (FA) = 90°. To obtain high SNR (temporal SNR in the spinal cord mask > 15) while allowing a high in-plane resolution, the spinal cord subvolume parameters were selected as follows: FOV = 132 × 132 mm^2^; in-plane resolution = 1.2 × 1.2 mm^2^; slice thickness = 5 mm with 4-mm gap (between the edges of adjacent slices); TE = 33 ms; bandwidth = 1,262 Hz/Px; and echo spacing = 0.9 ms. Parallel imaging using GRAPPA with an acceleration factor of 2 and 7/8 partial Fourier encoding were used for both subvolumes. Additional fat saturation pulses were applied. The total TR was 3,050 ms for the acquisition of 33 brain and 10 spinal cord slices. To reduce noise, only the signal from the head (or neck) coil elements was considered for the reconstruction of the brain (or spinal cord) slices. Additionally, 2 saturation pulses were applied in a V-shaped configuration to minimise ghosting and inflow artifacts related to blood flow in the major cervical vessels (Fig 1K). Also, flow rephasing gradient pulses were applied in slice direction to minimise signal variations related to CSF [14].

#### Shimming procedure

A dynamic update of the resonance frequency and the linear shims [14] was used during EPI measurements in order to optimise shim adjustment parameters for each of the brain and the spinal cord FOVs. These parameters were calculated in a “shim procedure” that took about 25 minutes to perform and took place before the start of functional scans. During this time, EPI volumes were acquired to calculate optimal linear shim and resonance frequency values for each of the brain and spinal cord FOVs, as well as a combined large FOV consisting of both the brain and spinal cord (Fig 1K). As the second-order shim values could not be dynamically updated in our MR system, they were calculated based on the previously mentioned combined FOV to obtain good quality images of both the brain and spinal cord.

Afterwards, in order to compensate for through-slice dephasing effects, a slice-specific (z-shim) gradient momentum was used for the spinal slices [15]. This procedure took approximately 10 minutes extra; during this time, a set of images, consisting of the spinal cord images with 21 equidistant gradient steps for each slice, was acquired prior to the functional scan. Subsequently, the gradient setting was selected, for each slice, that yielded maximum signal intensity within the spinal cord.

#### Structural scans

A 3D-MPRAGE sequence was used to acquire T1-weighted anatomical images covering the entire head as well as neck down to the T3 vertebral level using the following parameters: FOV = 175 × 264 × 384 mm^3^; sagittal slices; TR = 2,230 ms; TE = 3.95 ms; FA = 7°; TI = 1.1 seconds; and resolution = 1 × 1 × 1 mm^3^. In addition, a multi-echo data image combination (MEDIC) sequence was used to acquire a T2*-weighted anatomical image covering the same target spinal FOV as in the spinal fMRI acquisition with identical centre position and orientation using the following parameters: transversal slices = 22; slice thickness = 5 mm (no gap); in-plane resolution = 0.6 × 0.6 mm^2^; TR = 50 ms; TE = 5.02, 9.87, 14.72, 19.57, and 24.42 ms; FA = 7°; bandwidth = 260 Hz per pixel; and GRAPPA acceleration factor = 2.

### Resting-state scan

Functional scans under resting-state condition were acquired using the simultaneous brain–spinal cord EPI sequence (parameters described earlier), while participants stayed awake, kept their eyes open (as checked by a video camera monitoring their face), and remained still. The resting-state scan took 7 minutes and 53 seconds (155 volumes). Resting-state scan was acquired following the acquisition of the T1-wighted structural image and the shim procedure but before the second structural scan (T2*-weighted MEDIC sequence), as described earlier.

### Image processing

fMRI images related to the brain and the spinal FOVs were stored as two separate DICOM files and were converted into the NIFTI format. Image processing was done separately for the brain and the spinal cord using the Spinal Cord Toolbox (SCT; version 3.1.1) [44], FSL (release 5.0) [45], and in-house MATLAB programs. Details of preprocessing and analysis are given subsequently.

#### Structural image preprocessing

For each participant, the C2 (second cervical) and T1 (first thoracic) segments of the cord on the T1-weighted image (Fig 1C) were identified through visual inspection, and their coordinates were used to initiate the segmentation process. The T1-weighted segmentation output was subsequently improved by smoothing the cord using the output of the first segmentation step and applying a second segmentation to the smoothed image. The result was inspected visually and corrected manually if needed. Then, a specific spinal vertebral level was selected visually (here C7), and its coordinates were used to label different vertebral levels (Fig 1J) for registration to the template (MNI-Poly-AMU, T2-weighted image, Fig 1D). The output was again checked visually, and the last two steps were repeated if needed. The resulting warping field was saved and used for future registrations (Fig 1I).

Similar to the T1-weighted preprocessing, the segmentation of the T2*-weighted MEDIC structural image (Fig 1B) was done in 2 steps, and the output was visually inspected and corrected if needed. Then, the warping field needed for the co-registration of the T2*-weighted and T1-weighted images was computed using the SCT registration tools (Fig 1H). Then, by multiplying the warping fields from the T2*-weighted to T1-weighted space (Fig 1H), and from T1-weighted to the template (Fig 1I), we obtained the transformation between the T2*-weighted image and the template. This transformation was further improved by the segmentation of the grey matter in the spinal cord using a dictionary approach [46] and multiplying a corrective local warping field (Fig 1G). This final transformation maps the image from the T2*-weighted space to the template by considering the grey matter structure in the spinal cord.

Finally, we computed the parameters needed to co-register the EPI image (Fig 1A) and T2*-weighted image. The resulting transformation (Fig 1F) was then combined with the grey matter corrected transformation from the T2*-weighted space to the template as described earlier, to obtain the final transformation from the EPI to the template (Fig 1E). Note that, in every step, we also calculated an inverse transformation from the destination to the source image, so we also obtained the transformation from the template to the EPI image by multiplying the inverse transformations described earlier.

### Spinal cord fMRI preprocessing

The first two volumes of fMRI data were removed to account for the time to reach equilibrium magnetisation. Next, motion correction was performed using the sct_fmri_moco function from SCT. The output was visually inspected, and motion correction parameters were updated if needed. Next, spinal cord segmentation was performed on the motion-corrected mean image using SCT. The output was inspected visually and corrected manually if needed (all manual corrections were performed by one person, and a second person examined and annotated the first person’s work to improve the output of this step and reduce variability). Next, the slice-timing correction was performed on the fMRI data. We used the brain reference slice for this procedure so that all spinal cord slices were time-corrected to align with the brain slices acquired in each TR. Then, time series data were temporally filtered using high-pass filtering at 0.01 Hz. The spinal cord segmentation mask was then used for registration between the fMRI and T2*-weighted image as described earlier.

### Brain fMRI preprocessing

Brain image preprocessing was performed using the FSL software package [47], using the same pipeline as described previously [3,4]. In summary, the skull was stripped using optiBET algorithm [48]. Two first volumes were removed. Motion correction (FSL, MCFLIRT) and slice-timing correction were performed, followed by high-pass temporal filtering (0.01 Hz). Finally, a Gaussian kernel of 5-mm full width at half maximum (FWHM) was applied for spatial smoothing. A linear affine (6 degrees of freedom) transformation registered the functional data to the T1-weighted anatomical space, followed by a nonlinear registration (FNIRT, FSL) [49] from the T1-weighted image to the MNI template (MNI-152-2mm). These two transformations were concatenated to register the brain fMRI data to the MNI space.

We used PNM (FSL) [50] based on the RETROICOR method [51]. This method models cardiac and respiratory related artifacts by calculating the cardiac and respiratory phases relative to each volume and slice in the fMRI time series. Based on this phase information, a low-order Fourier expansion is then calculated to model the effects of cardiac and respiratory processes. As suggested previously [12], we included 3 cardiac and 4 respiratory harmonics and 1 multiplicative term for the interactions between cardiac and respiratory noise, resulting in 18 slice-specific regressors per resting-state run.

### Functional connectivity analysis

We used both seed-based and ICA approaches to examine the functional connectivity between the brain and spinal cord areas during resting-state periods.

For the seed-based analysis, we first defined 2 sets of paired ROIs in the spinal cord. We used the MNI-Poly-AMU template to extract the segmented masks for the dorsal and ventral horns, as well as the left and right sides of the spinal grey matter, spanning the C5 to T1 spinal levels (Fig 1L). We then projected each of these masks to the functional space of each participant, using the computed inverse transformation between the template and the EPI image as described previously (Fig 1M–1O). To increase the SNR, we calculated the average BOLD signal in each mask in all of the 10 slices covering the C5 to T1 spinal levels. This ensured that we had enough voxels for averaging inside each mask. In addition to the 2 pairs of ROIs described earlier, we also defined separate masks for each quadrant in the spinal cord grey matter, resulting in left ventral, right ventral, left dorsal, and right dorsal ROIs. We then calculated the average spinal cord BOLD signal inside each quadrant for each resting-state run.

The spinal cord ROI time series were then entered in a GLM as the regressor of interest to identify brain areas whose activity correlated with each spinal cord ROI during resting-state periods. We included the time derivative of each ROI’s signal as a regressor in the GLM to account for possible time differences in the haemodynamic response function (HRF) of different cortical areas, as well as the latency for signal propagation from one cortical area to another [4]. Furthermore, in order to account for the effects of physiological noise in the BOLD signal, we included the 18 slice-wise PNM regressors (see earlier) as confound in the GLM model. We also calculated and included the following time series in the GLM model as confound: the mean white-matter BOLD signal, the mean CSF signal, and 6 motion correction parameter time series (x, y, and z translations and rotations derived from the motion correction step in preprocessing). The white matter and CSF masks were defined using the segmentation masks extracted from the T1-weighted image (FAST, FSL) and then registered to the functional space. For each participant and each ROI, a separate GLM analysis was performed using the FEAT tool in FSL. Time series statistical analysis was carried out using FILM (FSL) with local autocorrelation correction [49]. This analysis produced maps of all voxels that were positively or negatively correlated with an ROI’s mean time course. This was followed by between-individual analyses that were carried out using a mixed-effects model (FLAME, FSL [47]) on the contrast of parameter estimate and its variance images registered to the MNI space. This group-level GLM estimated the mean functional connectivity with the ROI time series averaged across all participants. All group-level statistical maps were then corrected for multiple comparisons using GRF theory, z > 2.3, and cluster-level threshold *p* < 0.05.

Furthermore, to specifically examine the activation volume in each brain hemisphere correlated with the left and right spinal hemicords, we identified all brain voxels with significant functional connectivity (*p* < 0.01) to each spinal ROI in each participant. Brain hemisphere masks were extracted from the MNI template brain mask, excluding the cerebellum. We then measured the activation volume in each brain hemisphere correlated to each spinal ROI. From this, we defined the following laterality index for each spinal hemicord and each participant:

$$Laterality\;index=\frac{Left\;active\;volume-Right\;active\;volume\;}{Left\;active\;volume+Right\;active\;volume\;}$$

Hence, a positive laterality index for a given spinal ROI means that more voxels were correlated with that ROI in the left brain hemisphere than the right hemisphere, and vice versa. The laterality indices were calculated for each participant, and the group averages were then evaluated.

Also, in order to identify brain areas that were specifically correlated with one (e.g., the left) spinal cord ROI but not with a second (e.g., the right) ROI, we performed an additional analysis in which both ROI time series were entered in a single individual-level GLM, and they were orthogonalized with respect to each other (results reported in S1 and S4 Figs). By using this approach, the shared variance between the two regressors is not attributed to either, and the corresponding statistical map to each regressor represents brain areas that are exclusively correlated with that regressor and not the other one (see [3] for more details). Following that, a similar group-level GLM analysis as described earlier was employed to obtain the mean statistical map across all participants.

### Joint ICA

In order to identify the relationship between the well-characterised brain RSNs (see [21] for a review) and the spinal cord resting-state activity, we used ICA by combining both the brain and spinal cord functional data in a joint ICA run. The brain fMRI data were preprocessed using the same preprocessing steps as described earlier, followed by registration to the MNI 2-mm space. Likewise, the preprocessed spinal cord fMRI data (as described earlier) were used for this analysis, followed by the registration to the MNI-Poly-AMU space and down-sampling to 1.2 × 1.2 mm^2^ in-plane resolution. Only voxels inside the MNI brain mask and the MNI-Poly-AMU spinal cord mask were retained and used for this analysis. Note that slice timing correction was performed as a preprocessing step to adjust slight time differences between the brain and spinal cord slices in each acquisition volume. The brain and spinal cord four-dimensional data were reshaped to each form a two-dimensional matrix with the size of *n*_T_ × *n*_B_ and *n*_T_ × *n*_S_, respectively, in which *n*_B_ and *n*_S_ represent the number of voxels in the brain and spinal cord masks, respectively, and *n*_T_ is the number of time points. Then, for each participant, the brain and spinal cord matrices were concatenated column-wise, to construct a combined two-dimensional matrix with the size of *n*_T_ × (*n*_B_ + *n*_S_). Afterwards, as a common preprocessing step in spatial ICA [52], principal component analysis (PCA) was performed to reduce the dimension of each participant data from *n*_T_ to 40 (40 was selected to retain at least 90% of data variance in each participant), hence resulting in a 40×(*n*_B_ + *n*_S_) matrix for each participant. For group analysis, we used a time-concatenation approach [53], by concatenating the data from different participants row-wise and constructing a large matrix of (40 * *n*_sub_) × (*n*_B_ + *n*_S_), in which *n*_sub_ is the number of participants. The FastICA algorithm [54] was used to extract 40 group-level joint brain/spinal cord components using parameters as reported elsewhere [52].

Out of 40 components, we identified 15 components that met the following criteria, matching the consistently reported brain RSNs in the literature [21]. Specifically, for each participant and component, we calculated the power spectrum related to the corresponding component’s time series (using Welch’s spectral density estimation method) within and outside the neural activity-related frequency band of the resting-state BOLD signal (0.01 to 0.1 Hz [2]). Then, those components that showed 4-times-greater average power within than outside this frequency range were selected and were visually checked to ensure that they do not represent physiological/scanner artifacts [55]. Seventeen out of 40 components passed the power threshold criterion. Of these, 2 were excluded because they were related to motion artifacts around the edges of the brain and ventricles. The remaining 15 components were retained, and their corresponding group-level brain and spinal cord z-score statistical maps were constructed. Both the brain and spinal cord group-level ICA maps were then thresholded at z > 3.1 and corrected for multiple comparison using GRF, cluster-level threshold *p* < 0.05 (using *cluster* tool, FSL software [49]).

## Supporting information

S1 Data Numerical values related to the graphs plotted in Figs 2 and 3B, as well as z-score values and MNI coordinates of activation peaks related to the statistical maps reported in Figs 2 and 3A, S1, S2, S3, S4, S5 and S6 Figs. MNI, Montreal Neurological Institute.
(XLSX)

S1 Fig The spinal hemicords are preferentially connected to the contralateral cerebral cortex at rest. The left column shows the location of spinal ROIs, and on the right their associated brain functional connectivity maps are presented. The left spinal cord (top row) is significantly correlated to the brain sensorimotor areas in the right hemisphere, while the right spinal cord (bottom row) is significantly correlated to the brain sensorimotor areas in the left hemisphere. In this analysis, the right and left ROIs are entered in a single GLM. Display conventions are as in Fig 2. GLM, general linear model; ROI, region of interest
(TIF)

S2 Fig Brain resting-state functional connectivity maps associated with the ventral (motor) spinal quadrants. Top and bottom rows show the brain areas that are significantly correlated with the right ventral and left ventral quadrants, respectively. Display conventions are as in Fig 2.
(TIF)

S3 Fig Brain resting-state functional connectivity maps associated with the dorsal (sensory) spinal quadrants. Top and bottom rows show the brain areas that are significantly correlated with the right dorsal and left dorsal quadrants, respectively. Display conventions are as in Fig 2.
(TIF)

S4 Fig Brain resting-state functional connectivity maps exclusively associated with each spinal cord quadrants. Each row shows the brain areas that are significantly correlated with different spinal quadrants at rest, including right ventral, left ventral, right dorsal, and left dorsal ROIs. In this analysis, the right and left quadrants are entered in a single GLM, resulting in one model for the ventral quadrants and one model for the dorsal quadrants. Display conventions are as in Fig 2. GLM, general linear model; ROI, region of interest.
(TIF)

S5 Fig Brain resting-state functional connectivity maps associated with the grey commissure of the spinal cord. Left shows the location of the spinal ROI in green, and on the right the associated brain functional connectivity maps are presented. The grey commissure is significantly correlated to bilateral brain areas including putamen, pallidum, caudate, thalamus, insula, and secondary somatosensory cortex. Display conventions are as in Fig 2. ROI, region of interest.
(TIF)

S6 Fig Independent components with small or no spinal cord cluster (spinal cord activation volume < 0.15 cm^3^, or 20 voxels). These brain networks include the right and left executive control (A, B), anterior (C), and posterior (D, G) parts of the default mode network, the auditory (E), and the medial and lateral visual networks (F, H). Display conventions are as in Fig 4. Color-coded activation maps indicate z-score values and are corrected for multiple comparisons using GRF, *p* < 0.05. GRF, Gaussian random field
(TIF)

## Abbreviations

BOLD: blood-oxygen–level dependent
CNS: central nervous system
CSC: cervical spinal cord
CSF: cerebrospinal fluid
EPI: echo-planar imaging
FA: flip angle
fMRI: functional magnetic resonance imaging
FOV: field of view
FWHM: full width at half maximum
GLM: general linear model
GRF: Gaussian random field
HRF: haemodynamic response function
ICA: independent component analysis
MEDIC: multi-echo data image combination
MNI: Montreal Neurological Institute
PCA: principal component analysis
PNM: physiological noise modelling
ROI: region of interest
RSN: resting-state network
SCT: Spinal Cord Toolbox
SNR: signal-to-noise ratio
TE: echo time
TR: repetition time
