# Supplementary figures and images for: Resting-state brain and spinal cord networks in humans are functionally integrated
Source: PLoS Biol. 2020 Jul 2;18(7):e3000789. doi: 10.1371/journal.pbio.3000789 (PMC7363111; doi:10.1371/journal.pbio.3000789)

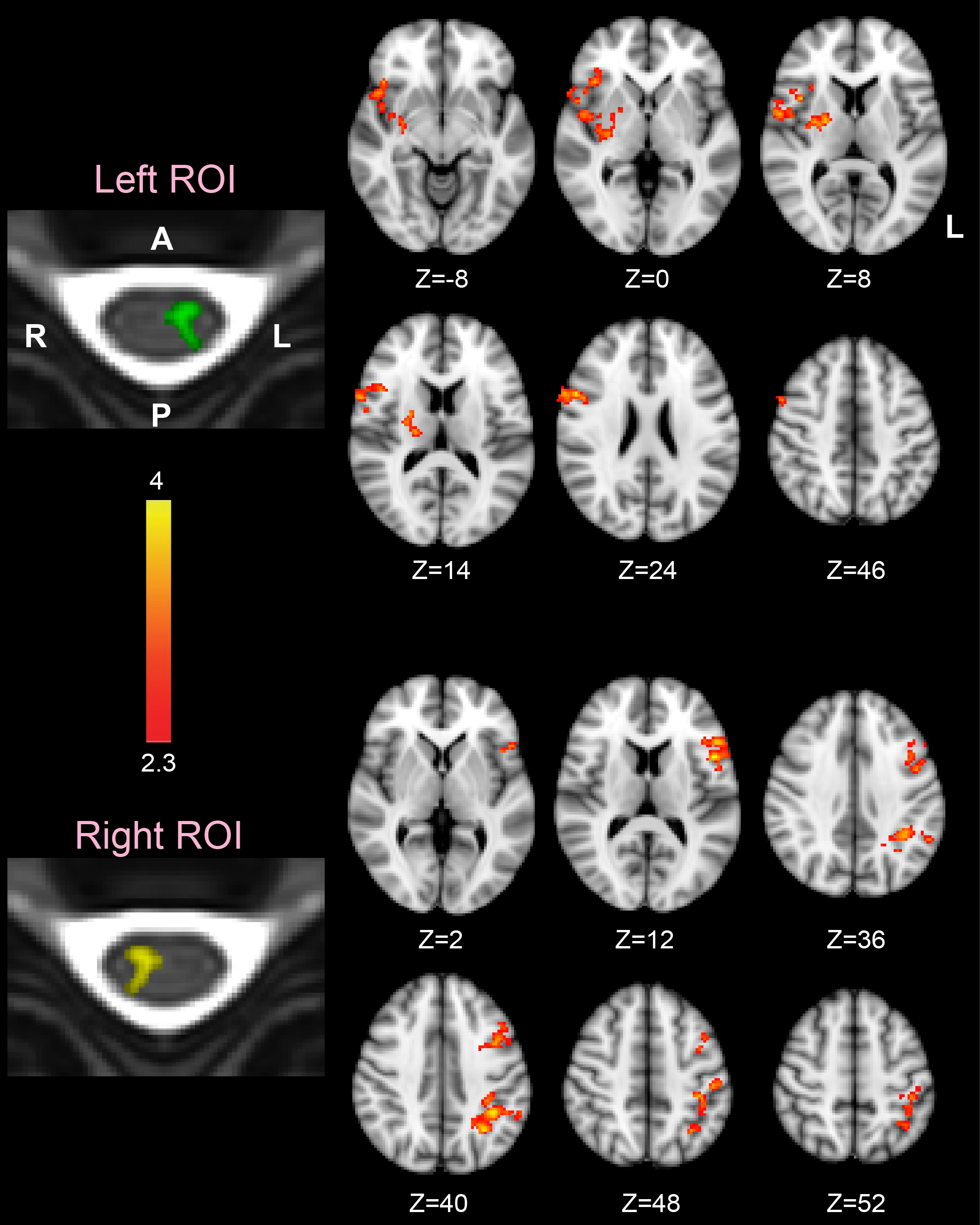

Supplement: S1 Fig — The left column shows the location of spinal ROIs, and on the right their associated brain functional connectivity maps are presented. The left spinal cord (top row) is significantly correlated to the brain sensorimotor areas in the right hemisphere, while the right spinal cord (bottom row) is significantly correlated to the brain sensorimotor areas in the left hemisphere. In this analysis, the right and left ROIs are entered in a single GLM. Display conventions are as in Fig 2. GLM, general linear model; ROI, region of interest (TIF) [file pbio.3000789.s002.tif]

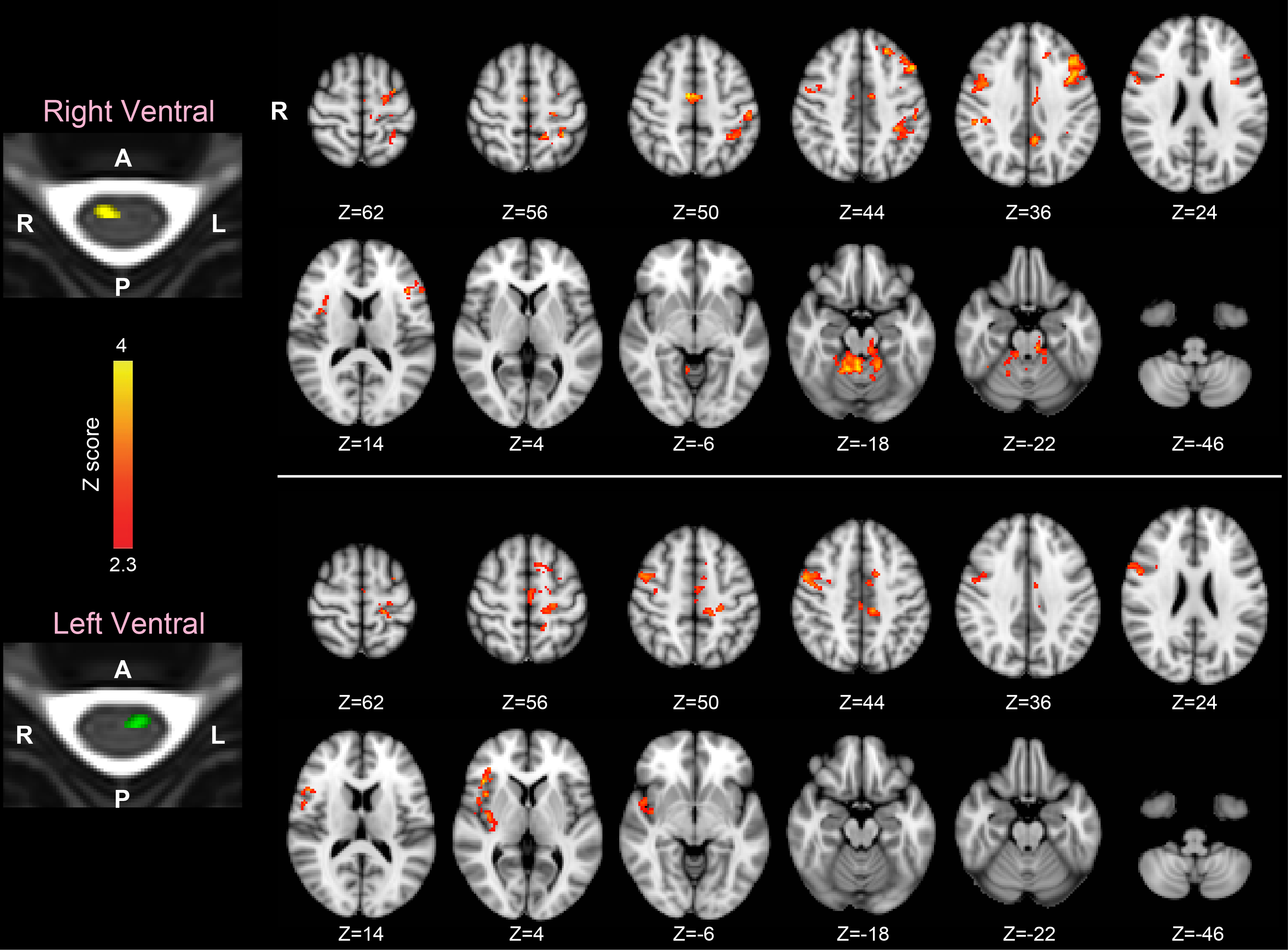

Supplement: S2 Fig — Top and bottom rows show the brain areas that are significantly correlated with the right ventral and left ventral quadrants, respectively. Display conventions are as in Fig 2. (TIF) [file pbio.3000789.s003.tif]

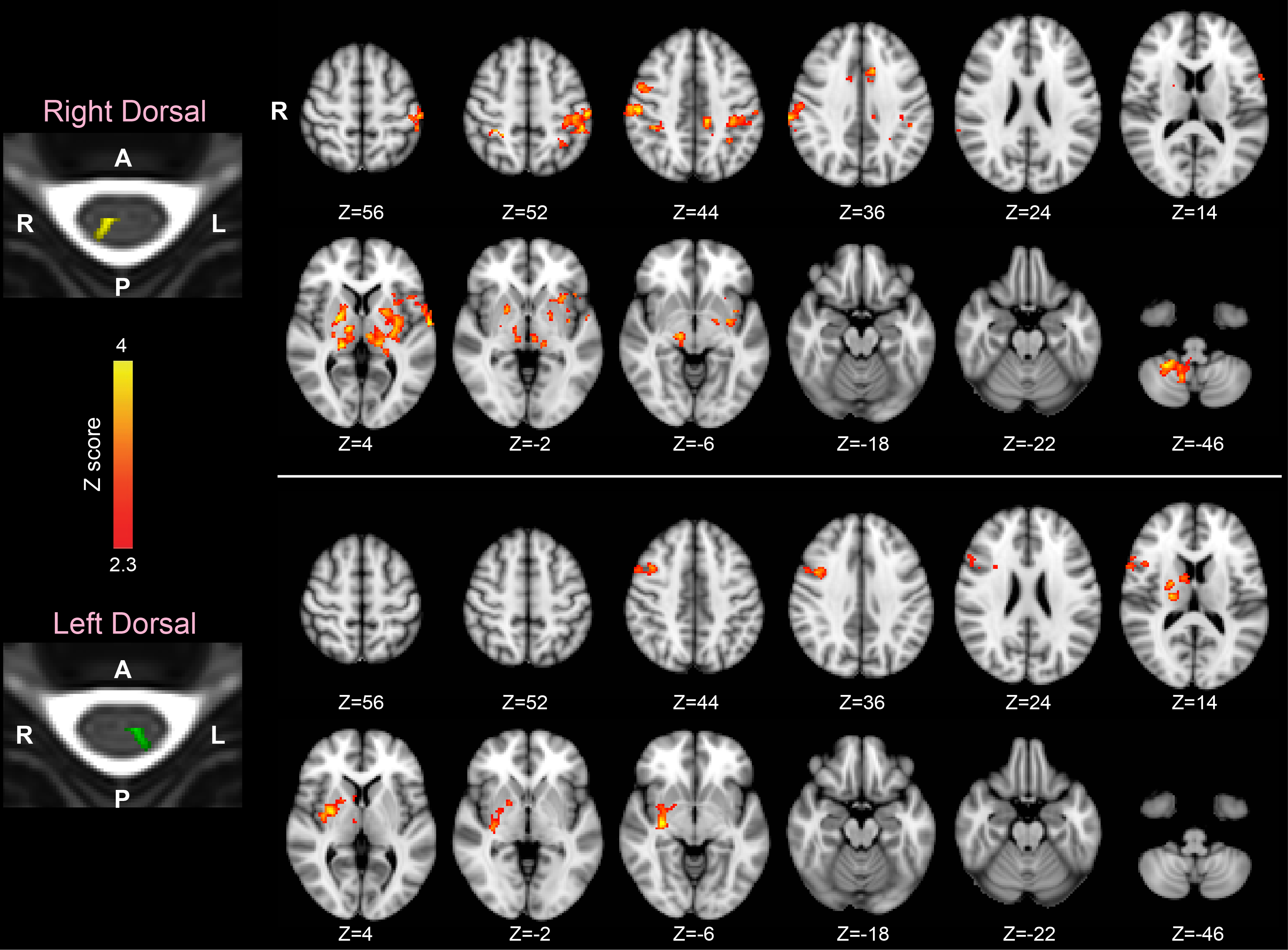

Supplement: S3 Fig — Top and bottom rows show the brain areas that are significantly correlated with the right dorsal and left dorsal quadrants, respectively. Display conventions are as in Fig 2. (TIF) [file pbio.3000789.s004.tif]

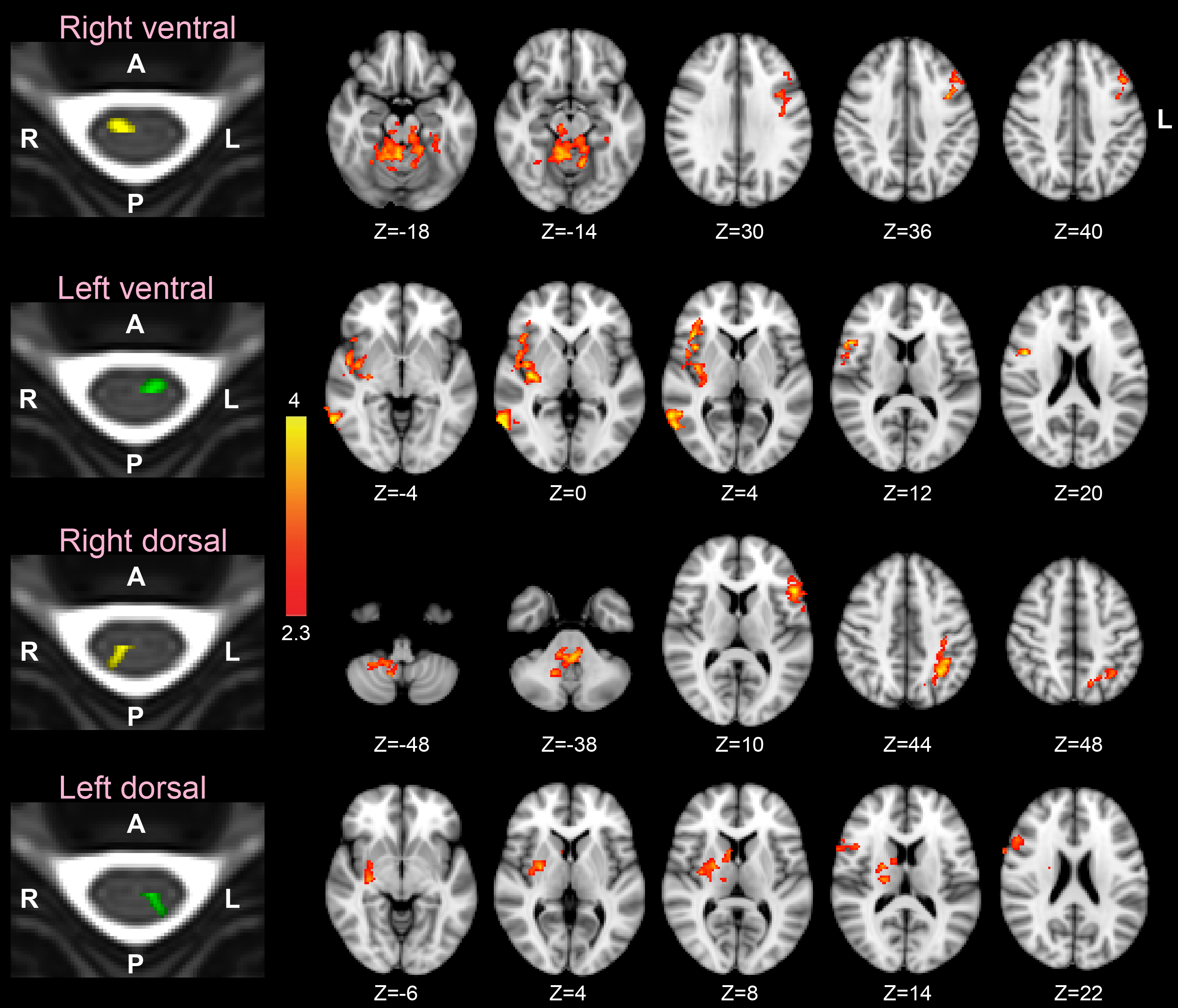

Supplement: S4 Fig — Each row shows the brain areas that are significantly correlated with different spinal quadrants at rest, including right ventral, left ventral, right dorsal, and left dorsal ROIs. In this analysis, the right and left quadrants are entered in a single GLM, resulting in one model for the ventral quadrants and one model for the dorsal quadrants. Display conventions are as in Fig 2. GLM, general linear model; ROI, region of interest. (TIF) [file pbio.3000789.s005.tif]

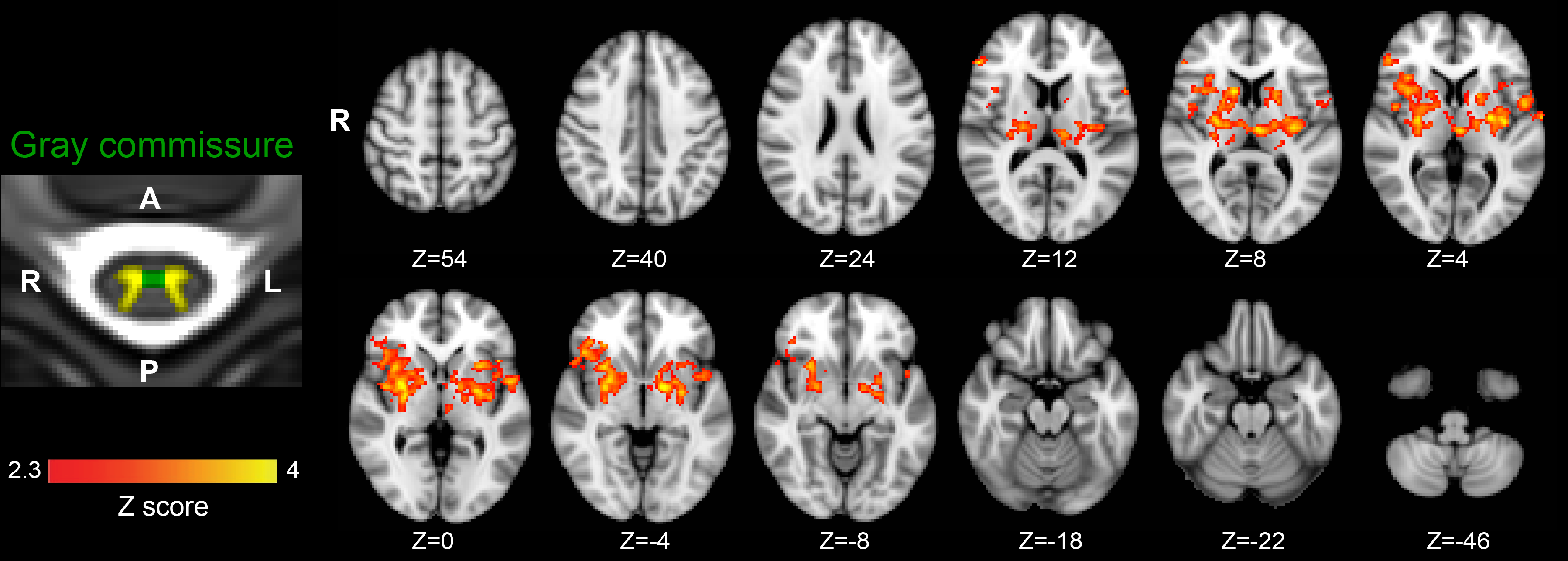

Supplement: S5 Fig — Left shows the location of the spinal ROI in green, and on the right the associated brain functional connectivity maps are presented. The grey commissure is significantly correlated to bilateral brain areas including putamen, pallidum, caudate, thalamus, insula, and secondary somatosensory cortex. Display conventions are as in Fig 2. ROI, region of interest. (TIF) [file pbio.3000789.s006.tif]

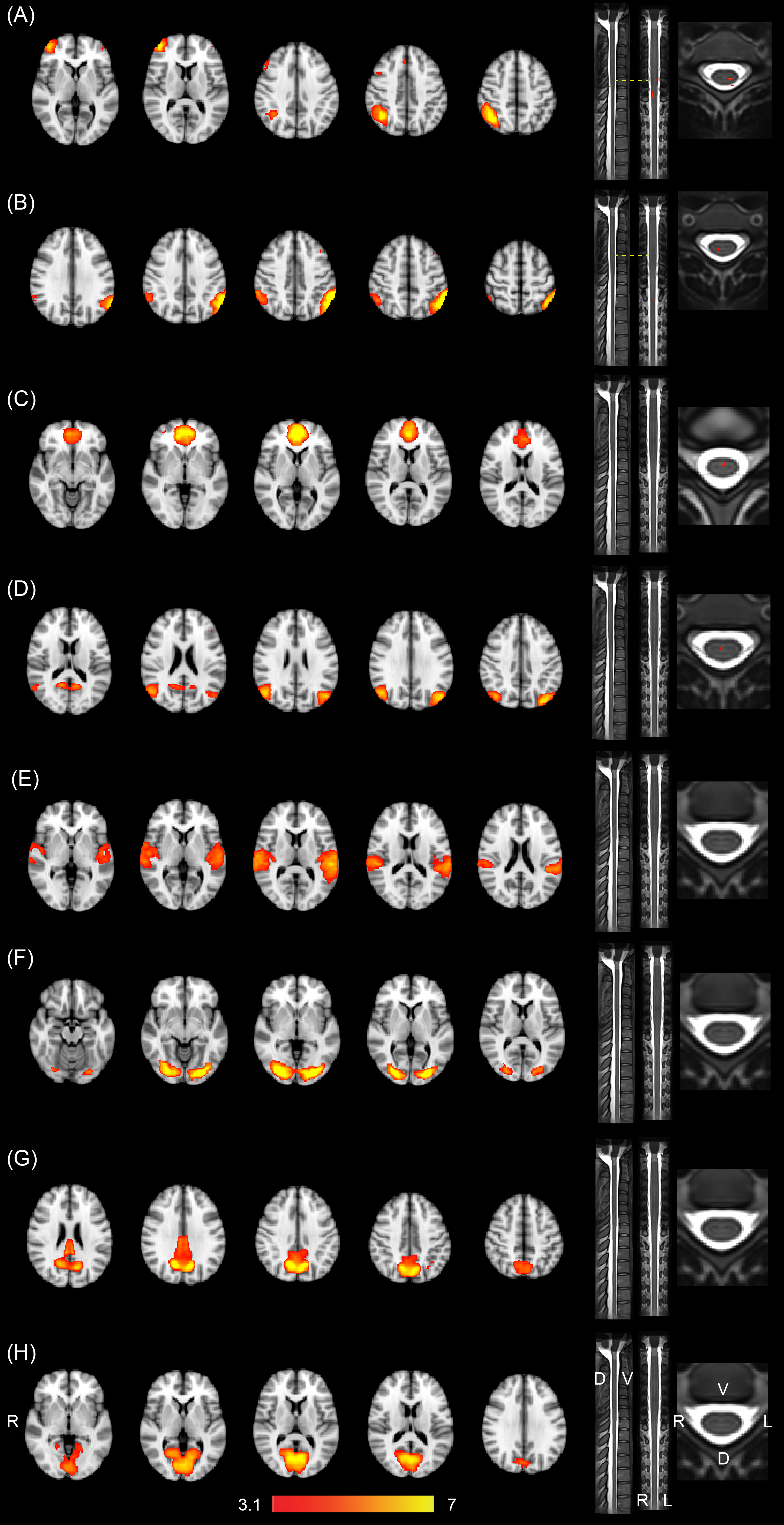

Supplement: S6 Fig — These brain networks include the right and left executive control (A, B), anterior (C), and posterior (D, G) parts of the default mode network, the auditory (E), and the medial and lateral visual networks (F, H). Display conventions are as in Fig 4. Color-coded activation maps indicate z-score values and are corrected for multiple comparisons using GRF, p < 0.05. GRF, Gaussian random field (TIF) [file pbio.3000789.s007.tif]
